# Book Reviews

**Published:** 1806-12

**Authors:** 


( To be continued. )

### CRITICAL ANALYSIS

OF THE

### RECENT PUBLICATIONS

ON THE

### DIFFERENT BRANCHES OF PHYSIC, SURGERY, AND MEDICAL PHILOSOPHY.

*Practical Observations on the principal Diseases of the Eyes, illustrated with Cases.* Translated from the Italian of ANTONIO SCARPA, Professor of Anatomy at the University of Pavia, F. R. S. Lond. and Member of several other learned Societies, with Notes. By JAMES BRIGGS, Member of the Royal College of Sugeons of London, and Assistant Surgeon to the Public Dispensary. 8vo. London, 1806.

WE were much surprised at a preface by the translator, in which he objects to the division of the practice of surgery like that of other arts. We are ready to admit that, in order to acquire a competent knowledge of diseases of the eye, it is necessary to be well acquainted with the principles of chirurgical physiology and anatomy. But there there is much difference between practice and study; and though, without doubt, every person who undertakes the treatment of a single disease should be regularly educated, yet, by confining his practice to a single object, he cannot but become better informed in whatever relates to it. In the operative branches of surgery, this is particularly desirable: because practice only can render the operator cool under, and prepared for, every difficulty, as well as ready under all circumstances. In the nicer operations, this is particularly necessary. Nothing but frequent early opportunities can, in our opinion, fit a person advanced to the full strength of his judgment, to undertake the extraction of the cataract.

The remainder of the preface gives an account of the manner in which the translation has been conducted; every part of which will be approved by the reader.

The author's preface follows, which relates principally to the manner in which he has conducted the work, and his reasons for undertaking it.

The first Chapter is of the puriform discharge from the palpebræ, and of fistula lachrymalis. The exordium gives us a very favourable impression of the professor's accuracy. He distinguishes between a small quantity of pus, which appears at the puncta lachrymalia, and the true fistula lachrymalis. The first he very properly calls a puriform state of the via lachrymalis,* arising from such a discharge along the palpebræ, which finds its way to the orifice of the duct, and may be pressed back. This may give a suspicion that the duct itself is diseased, and the surgeon may have the credit of curing fistula lachrymalis with very little trouble. The true fistula lachrymalis is next described, and the mode of treatment is very judicious. Some of the remarks may be called new, even in this country, where the subject has been so often and so nicely examined. Some useful cases are added to illustrate the whole.

The second Chapter is on the hordeolum, or stye. This the author distinguishes from the small abscess, which sometimes occurs in those parts, by remarking, that the contents, instead of being purulent, consist wholly of dead matter. The mode of treatment in its different stages, is such as is familiar with most practitioners.

The third Chapter comprehends incysted tumours of the eyelids. This is a very important subject, and we cannot help admiring the candour of the author, in acknowledging that he has never been able to resolve these tumours by any of those applications which have been so much extolled by others; nor, indeed, by any other means, after they had arisen to such a size as to determine their real character. He therefore considers, that extraction is the only remedy, which should be attempted as early as possible,

O o 2 that

---

* In the translation it is "via lachrymalia." We know not how such an inaccuracy of the printer could have been overlooked.

that the operation may be the more easy to the patient. The reader will recollect, that this impossibility of treating such tumours, in the same manner as any other diseases, induced Dr. Adams to consider them as possessing all the properties of hydatids, in consequence of which the eyst, having an œconomy peculiar to itself, could not partake of those actions by which diseased parts, particularly the capsules of abscesses, are absorbed, or so altered as to return to their former organization. The professor, with much judgment, contents himself with stating the facts as they have occurred, without engaging in the controversies of others, or any theories of his own.

Chapter IV. is of the cilia, which irritate the eye. This chapter contains much new matter, related with an accuracy such as we might expect from the author.

"This disease, which is termed trichiasis, presents itself under two distinct forms: the first is where the cilia are turned inwards, without the tarsus having changed its natural position and direction; the second consists in a morbid inclination of the tarsus, and consequently of the eye-lash towards the ball of the eye.

"The first form of this disease is very rare, nor has it come under my own observation more than once, and in this instance only some of the hairs had changed their direction. The second species or form of *trichiasis*, or that which consists in a folding inwards of the tarsus and cilia at the same time, is that which is commonly met with in practice. This may be either complete, affecting the whole of the tarsus; or incomplete, occupying only a certain portion of the edge of the eye-lid, and most frequently near the external angle of the eye; sometimes the disease is confined to one eye-lid only, at other times it affects both, and occasionally the patient is afflicted with it in both eyes.

"To these two species of *trichiasis* some writers have added a third, which they call *distichiasis*, and which they suppose to be produced by a double and unusual row of hairs. But this third species is only imaginary, and the reason of such subdivisions seems to have arisen from a want of recollecting what was long ago remarked by Winslow* and Albinus† on the natural arrangement of the cilia; that although their roots appear to be disposed in one line only, they nevertheless form two, three, and in the upper eye-lid even four ranges of hairs, unequally situated, and as it were confused. Whenever, therefore, in consequence of disease a certain number of hairs are separated from each other in a contrary direction and disorderly manner, the eye-lash will appear to be composed of a new and unusual row of them, while in fact there has

---

* Exposition Anatom. Trait. de la tête, § 278.

† Acad. Annotat. lib. iii. cap. 7.

has been no change either with respect to their number or natural implantation.*

"It is not an easy matter to determine precisely what are the causes which sometimes occasion a small number of the hairs to deviate from their natural direction, while the tarsus remains in its position. They are generally attributed to cicatrices which take place upon the tarsus in consequence of previous ulceration, by which the cilia fall off, and those which are naturally growing are prevented from taking their proper direction. But it is proper to remark, that this cause is not the only one, since in the case which occurred to me, two or three hairs were turned inwards against the eye-ball, although there had been neither ulceration, nor cicatrization of any part of the tarsus.

"For my own part, I am inclined to think, that the small ulcers and cicatrices, which are occasionally formed on the internal margin of the tarsus, rather give rise to the second form of the disease, or the inversion of the edge of the eye-lid, and consequently of the cilia towards the ball of the eye. As these ulcers are of a corroding nature, and when neglected destroy the substance of the internal membrane of the palpebræ near the tarsus, it necessarily follows, that in proportion as they heal and contract themselves, they draw along with them and turn inwards the tarsus, and consequently the hairs which are implanted in it. And as these small ulcers do not always occupy the whole extent of the internal margin of the eye-lid, but are sometimes confined to a few lines in the middle or extremity, near the external angle of the eye-lid, so after the cicatrices are formed, the whole of the hairs are not always turned inwards, but only a certain number of them which correspond to the extent of the ulcers previously situated along the internal margin of the tarsus. Indeed, in every case of imperfect trichiasis, in consequence of a cicatrix of the internal surface of the edge of the eye-lid, a very slight examination will show, that the tarsus and cilia are every where in their natural situation, except opposite the part where the ulcers had formerly existed; and if the eye-lid be everted, it will be evident that the internal membrane near that part of the margin corresponding to the seat of the trichiasis is pale, rigid, and callous, and that from this contraction the inversion of its cartilaginous border is evidently derived, as well as the morbid inclination of the hairs towards the globe of the eye.

"Besides these causes, there are others capable of producing the same injurious effect. In the first place the chronic ophthalmia of long standing, as that which arises from scrofula or the small-pox, which becoming gradually worse and worse, keeps the integuments of the eye-lid for a considerable time in a state of

* Maître-Jan made the same observation, a long time ago, as may be seen in his Traité des maladies de l'œil, p. 494.

distension and œdema, and induces a relaxation of them, by which the cartilaginous border of the eye-lid ultimately losing a proper and firm support in the integuments, inclines towards the eye-ball, and afterwards turns inwards, and draws the cilia along with it in the same improper direction. The same unpleasant effect, independently of the relaxation of the integuments, is frequently produced by a softening of the cartilage of the tarsus, in consequence of a copious and long continued puriform discharge from the ciliary glands, by which the cartilage of the tarsus becomes either wholly or partially incapable of supporting itself erect, or of preserving the curve necessary to its perfect coaptation with the tarsus of the other eye-lid: hence the cartilage, either in the whole, or a part of its extent, becomes relaxed and folded inwards, and draws along with it the corresponding hairs against the ball of the eye.

"These causes are not unfrequently found combined together, and they are also often accompanied with cicatrices of the membrane which invests the internal margin of the tarsus. Some * pretend that the trichiasis is occasionally produced by a spasmodic contraction of the orbicularis palpebrarum. But I must confess that this has never come under my own observation, and it is difficult to believe that the spasm of this muscle, however violent, can produce a folding inwards of the tarsus and cilia, much less that it should continue to act as a permanent cause of the disease.

"The degree of uneasiness which must necessarily result from the hairs perpetually pressing upon the cornea and white of the eye, may be easily calculated even by those who are little acquainted with surgery. To aggravate this evil still more, it very frequently happens, that the hairs bent inwards acquire a much greater length and thickness than those which retain their natural position. And although the disease be confined to one eye, yet from consent, both are usually affected, and the sound eye cannot be moved without occasioning pain in that which is subjected to the irritation and friction of the inflected hairs. In general, it may be said, that both the eyes in persons affected with this disease are very irritable and impatient of the light. As the patient, in cases of incomplete trichiasis, retains some little power of opening the eye-lids for the purpose of seeing, and that most frequently towards the internal angle of the eye, the head and neck are frequently inclined in an aukward manner, producing in children, at length, a distortion of the neck and shoulders, which is with difficulty corrected, even after the trichiasis is cured. Children besides, impatient of the irritation which the inflected cilia produce, are incessantly rubbing the eye-lids, which contributes in no small degree to increase the evils consequent on the trichiasis; such are the *varicose chronic ophthalmia*, the *nebula*, and the *ulceration of the cornea.*"

The

* Bell's System of Surgery, vol. iii. p. 276.

The cure proposed for the second species of Trichiasis is, by an excision of the under portion of the skin close to the edge of the eye-lid, so that the contraction formed by the future cicatrix may give the ciliæ a different inclination. The operation and subsequent treatment are well described, and the remedies against the other diseases explained with the same accuracy: the chapter concluding, like the rest, with some well selected cases. The next chapter, of the relaxation of the upper eye-lid, is equally judicious, the various causes well pointed out, and a proper distinction made between the disease when arising from a paralytic state, or too great elongation of the lid, and when only a symptom of other complaints. In the first case, the remedy proposed is similar to that for trichiasis.

Chap. VI. is of the eversion of the eye-lids. This disease, of course, arises from causes directly opposite to the former. The remedies proposed by the Professor are extremely simple, which is no inconsiderable recommendation, as they are not less judicious.

Chapter VII. begins the subject of Ophthalmia. This is divided into chronic and acute, and shows, with much force, the necessity of this descrimination, the remedies for each being diametrically opposite. The favourite remedy first recommended by Mr. Ware, the tinct. thebaica of the old pharmacopœia, is said to be not less injurious in the acute than serviceable in the chronic, or which is often the same thing, highly serviceable in the progress of a disease, which it has exasperated at the beginning.

On the subject of venereal ophthalmia, or ophthalmia by metastasis from gonorrhœa, our author speaks with much less decision than on most others, and we wish, for his own sake, that he had omitted it altogether. A judicious note is added by the translator, which his modesty has induced him to support by the authority of Mr. Pearson. As the subject is highly important, and stands here unconnected with any other, we shall transcribe the note.

"The reasons which have led Professor Scarpa to doubt the opinion of the particular manner in which the gonorrhœa produces this affection of the eyes, would also I think lead one to suspect the existence of such a cause altogether; but the following communication, for which, says Mr. Briggs, 'I am indebted to Mr. Pearson, forms a more satisfactory argument than any presumptive evidence that can be offered.

"The venereal ophthalmia, or what Professor Scarpa calls the gonorrhœal ophthalmia, whether ascribed to metastasis, sympathy, or the application of the matter of gonorrhœa to the eye, is a disease which has been described by a considerable number of those writers who have treated professedly on venereal complaints; but whether the greater part of them have given the result of their own observations, or have merely transcribed from the works of their predecessors. is a question deserving some consideration.

"Although I am fully disposed to treat the talents and accuracy of Professor Scarpa with the utmost deference, yet I cannot help entertaining some doubts of the propriety of assigning the gonorrhœa as a cause of ophthalmia; since, during a pretty extensive experience of twenty-five years, I have never seen one single instance of an inflammation of the eyes, which was evidently derived from a gonorrhœa. I am sufficiently aware of the nature and force of negative evidence in matters depending on testimony, not to over-rate it; and certainly, to deny the existence of any attested fact, merely because it has not occurred in the course of a man's own experience, would be hasty and unjustifiable. In the instance now before us, there are two points to be considered; the testimony of a respectable Professor, and the validity of his opinion; for it is not only asserted, that those who are infected with a gonorrhœa may be attacked by a violent ophthalmia, but that the gonorrhœa is somehow or other the cause of that ophthalmia. It is with reference to the latter proposition that I express my doubts, which are founded upon the fact mentioned before, that, of the many thousand cases of gonorrhœa which have fallen under my notice, I never could, in any one instance, trace such a connexion between the eye and the urethra, as that to which Professor Scarpa alludes.

"The puriform ophthalmia of infants, was, within my recollection, generally regarded as an indication of a venereal taint; and much unnecessary distress was often excited in families, and very improper treatment was frequently pursued in consequence of this erroneous opinion. The nature of that complaint, and the proper method of treating it, are now much better understood, and I conceive, that mistakes in these cases are not very common at this time.

"In that form of the secondary symptoms of syphilis, where the skin is the part chiefly affected, a disease resembling the ophthalmia tarsi sometimes appears. It is not commonly attended with much redness of the tunica conjunctiva, nor is the sensibility of the eye to light remarkably increased: yet I have seen it, in a few instances, in the form of an acute ophthalmia, resisting all the common modes of treatment, but yielding immediately to a course of mercury.

"The venereal ophthalmia resembles, in its appearance, those diseases of the tarsi and tunica conjunctiva, which are derived from scrofula; and, I believe, there are no specific characters by which diseases of the eye, or eye-lids, produced by the action of the venereal virus, can be distinguished from those which are excited by other causes."

( To be continued. )

*Observations on Indigestion, in which is satisfactorily shown the Efficacy of Ipecacoan, in relieving this, as well as its connected Train of Complaints peculiar to the Decline of Life.* Translated from the French Memoir of M. DAUBENTON, Member of the R. Med. Soc. Paris.

AN Introduction, by the translator, informs us, that the author was originally destined for medicine, but acquiring a fondness for natural history, by his acquaintance with the celebrated Buffon, by whom he was employed in his dissections for comparative anatomy, his whole attention was afterwards directed to those enquiries. An attention to his health, however, induced him to examine into the disease here treated of; and by great attention, he discovered a remedy, which he could have no motive in recommending, but public utility. It is further remarked, in favour of the proposed remedy, that the author lived to the age of eighty-four; and, lastly, that the translator has found every practical advantage he could expect, from the information contained in the work.

The work itself begins with a division of the life of man into various periods, marking, more particularly, those in which the different organs (particularly the stomach) begin to decline in the proper exercise of their functions. After this, we have a description of the effects of indigestion; among the rest, the evolution of air; to which, it is well known, that the French impute most of those diseases, which in England are now commonly called nervous. The various kinds of food are next enumerated, as they are subservient to the nourishment of the inferior animals and of man. Here we are informed, that the peasantry, if not obliged to labour too hard, and have sufficient clothing, enjoy health and strength, with a diet consisting principally, if not entirely, of vegetables; whilst the more wealthy, whose food is composed of every thing that they can obtain, though apparently in higher health, are subject to a variety of diseases, which exercise only can prevent or relieve. Even this, however, joined to a more sparing indulgence, will rarely be sufficient; so that, on the whole, the author conceives vegetable substances the proper food for man. His argument, on this occasion, is somewhat obscure; but as far as we can understand him, it appears his opinion, that it is necessary the stomach should be distended to a certain degree, and, that if this distention is occasioned by animal food, the consequence will be, a quantity of nutritious matter greater than the constitution can dispense with. It is not our intention to comment on this, or other physiological errors, which appear to us in the work; it is enough, that the remedy was found practically ueful, in which there is no reason to doubt the evidence of M. Daubenton, or his Translator.

---

The

*The Naval, Military, and Private Practitioner's Amanuensis Medicus et Chirurgicus: or a Practical Treatise on Fevers, and all those Diseases which most frequently occur in Practice, with the Mode of Cure; likewise on Amputation, Gun-shot Wounds, Trismus, Scalds, &c. with a new and successful Method of treating Mortification, of amputating at the Shoulder Joint, and of curing Femoral Fractures.* By Ralph Cuming, M. D. R. N. &c. London, 1806. Octavo, pp. 368.

The following is the arrangement adopted in the above work: Continued and intermittent fevers, ophthalmia, inflammatory sore-throat, croup, pneumonia, hepatitis, jaundice, rheumatism, lumbago and sciatica, gout, erysipelas, hæmoptysis, scurvy, piles, catarrh, influenza, dysentery, diarrhœa, lues venerea, gonorrhœa. — These subjects occupy a little more than half the volume; the remainder, which the author considers as more original, or containing more of novelty, comprises observations on amputation, on luxations, a discourse on fractures, on scalds and burns, in the treatment of which he prefers the refrigerating plan; on ulcers, gun-shot wounds, locked jaw, in the treatment of which he found opium dropped into the ears of essential service, and he suggests the propriety of trying the *tinctura ferri muriata* in doses of twenty drops every ten minutes.

The volume concludes with the treatment of sphacelus or mortification, which the author justly considers as the most original and interesting part of his work. The improvement consists in applying *nitre* finely powdered to the surface of the wound, as our readers have already seen in this Journal. The success of the application of nitre suggested to us the propriety of trying the oxygenated muriate of potash in the same manner. This hint has been given to several surgeons, but we have not yet received any account of its success.

We think Dr. C. has deserved well of the profession by this publication, and that surgeons in the army and navy in particular will find it well worthy of their attention.

---

*The American Dispensatory, containing the Operations of Pharmacy, together with the Natural, Chemical, Pharmaceutical, and Medical History of the different Substances employed in Medicine, illustrated and explained, according to the Principles of modern Chemistry; comprehending the Improvements in Dr. Duncan's second Edition of the Edinburgh New Dispensatory; the Arrangement simplified, and the Whole adapted to the Practice of Medicine and Pharmacy in the United States: with several Copper-plates, exhibiting the new System of Chemical Characters, and representing the most useful Apparatus.* By John Redman Coxe, M. D. &c. &c. Philadelphia, 1806. Octavo, pp. 800.

We are not less surprised, that America should have waited so long for a Dispensatory of its own, than we are pleased that this want has has been supplied so ably and judiciously by Dr. Coxe. The arrangement is alphabetical. All the useful tables of thermometers, specific gravity, solubility, affinity, &c. are given with great accuracy, and we congratulate the medical public on the acquisition of so correct and valuable a work.

